# Improving early detection of mental disorders & emergencies in resource-limited prison systems: development of a structured screening and risk stratification instrument in Indonesia

**DOI:** 10.3389/fpsyt.2026.1819710

**Published:** 2026-07-02

**Authors:** Natalia Widiasih Raharjanti, Corine de Ruiter, Adhitya Sigit Ramadianto, Monika Kristi Levania, Aisha Emilirosy Roekman, Claudia Gunawan, Dyta Ghezhanny William, Natasya Reina, Grady Krisandi

**Affiliations:** 1 Division of Forensic Psychiatry, Department of Psychiatry, Faculty of Medicine, University of Indonesia, Jakarta, Indonesia; 2 Faculty of Psychology and Neuroscience, Maastricht University, Maastricht, Netherlands

**Keywords:** inmate, mental disorders, prison, psychological well-being, screening

## Abstract

**Background:**

Mental health screening in prisons is vital to uphold prisoners’ right to mental health services, ensure safety, improve quality of life and reduce recidivism among incarcerated individuals. As frontline staff, correctional officers need a guideline for this screening to refer cases to the appropriate medical services. The currently available screening tool in Indonesia is unsuitable for detecting a number of relevant mental health issues and lacks sensitivity. Therefore, this study aimed to develop a new screening tool for mental disorders among inmates in Indonesia through an evidence-based approach.

**Methods:**

To develop a standardized screening tool for mental disorders in Indonesian inmates, we conducted a literature review, needs assessment survey, and Delphi-based expert consensus to identify gaps in Indonesia’s present tool, to refine and modify the domain items in line with the Diagnostic and Statistical Manual of Mental Disorders, 5th edition.

**Results:**

Six widely used screening tools were reviewed and the mental disorders screened were examined. Findings from the needs assessment survey and expert panel highlighted several key needs, including clearer operational definitions, more observable item indicators, integration of assessment methods, and the inclusion of structured risk stratification and emergency management flowcharts. We designed an eight-domain (depression, risk of suicide, anxiety, psychosomatic disorder, aggression, bipolar disorder, psychotic disorders, and psychiatric/medical drug consumption history) and 27-item screening tool to screen for mental disorders among inmates in Indonesia.

**Conclusion:**

The new 27-item screening tool was developed based on a review of the international research literature, needs assessment survey, and Delphi-based expert consensus. The tool demonstrated preliminary content validity through expert consensus and may serve as a structured screening framework for early identification of priority mental health conditions in Indonesian correctional settings. Further psychometric and field validation studies are required before routine implementation.

## Introduction

Mental health disorders represent a significant challenge in correctional settings worldwide (1). Within Indonesian prisons, the prevalence of these disorders is notably high, exceeding the 14.5% rate typically reported among general male inmate populations (2). Mental health disorders constitute a major public health concern in correctional settings worldwide. In Indonesian prisons, the prevalence of mental health disorders among inmates is particularly high, surpassing the 14.5% rate commonly observed in general male inmate populations. These challenges are compounded by structural limitations: despite the existence of approximately 531 correctional facilities nationwide, mental health infrastructure within prisons remains minimal, with limited psychiatric services, inadequate crisis management systems, and scarce trained personnel to respond to acute psychological emergencies) (3).

The inadequate management of mental health disorders within correctional settings significantly increases the likelihood of acute psychological crises such as suicide, institutional aggression, and interpersonal violence, posing risks not only to the affected individuals but also to the broader inmate population and correctional staff. Studies of inmate deaths in Indonesian prisons indicate that suicide represents a significant proportion of in-custody fatalities, accounting for approximately 20.8% of prisoner deaths in some reports, underscoring the urgent need for early mental health identification and intervention (4). Compounding this issue, inappropriate cell placement resulting from inadequate risk assessments often place vulnerable individuals in high-risk environments, further escalating the potential for violence and institutional instability. In Indonesia, this problem is exemplified by the co-housing of general inmates with terrorist prisoners due to overcrowding and the absence of specialized facilities. Such arrangements generate complex psychosocial environments marked by intimidation, ideological pressure, and heightened intergroup tension. Consequently, mixed placements complicate clinical and security risk assessment, increase psychological distress and institutional conflict, and impede accurate identification of mental health needs. For inmates with pre-existing vulnerabilities, exposure to high-risk or ideologically driven groups may accelerate symptom deterioration, undermine rehabilitation, and heighten safety risks for both correctional staff and the wider prison population.

When individuals with complex mental health needs are housed in settings misaligned with their clinical and security profiles, their conditions frequently deteriorate, undermining rehabilitative efforts, and contributing to higher rates of recidivism (1). As such, the early identification and comprehensive management of mental health disorders are increasingly recognized as critical components of effective correctional and rehabilitative strategies. However, implementation is frequently constrained by the limited availability of trained medical and mental health professionals within correctional institutions.

The STAIR model is a potential answer to such resource constraints (2). The STAIR model (Screening, Triage, Assessment, Intervention, and Re-integration) developed by Simpson et al. outlines a systematic approach to correctional mental health care, starting with trained staff conducting universal screenings with validated instruments to identify conditions requiring prompt intervention or specialist referral (2, 5).

A significant challenge in the Indonesian context is the prevailing perception among correctional officers that inmates are manipulative or prone to malingering, and therefore less likely to suffer from genuine mental health conditions (6). This bias is also observed in other countries which contributes to the underdiagnosis of mental disorders (7). Furthermore, there is a lack of dedicated mental health screening tools. Instead, mental health–related questions are embedded within broader assessment instruments, such as the *Instrumen Skrining Penempatan Narapidana* (ISPN) or Inmate Placement Screening Instrument (IPSI), *Sistem Penilaian Pembinaan Narapidana* (SPPN) or Inmate Rehabilitation Assessment System (IRAS), and *Risiko Residivisme Indonesia* (RRI) or Indonesian Recidivism Risk (IRR). Each of these tools serves a different purpose and is administered by different officers at various stages, impeding continuous evaluation of inmates’ symptoms, which may fluctuate over time. ISPN, used by probation officers, primarily assesses security needs and includes a few items related to aggressiveness; SPPN, used by correctional guardians, tracks rehabilitation progress through structured evaluations; and RRI is a pre-release recidivism risk assessment. However, reports of undetected mental health conditions in several Indonesian prisons indicate that the current screening system is insufficient (8, 9).

Obviously, adding mental health screening instruments presents an additional burden for correctional officers, many of whom already report emotional exhaustion and reduced sense of accomplishment, with around 37- 71% experiencing burnout symptoms (10). The lack of specialized mental health personnel further shifts responsibility to correctional officers, who often have limited specialized training and diverse educational backgrounds.

Recent legal reform further underscores the urgency of strengthening early mental health identification. Article 38 of Indonesia’s new Criminal Code (Kitab Undang-Undang Hukum Pidana, KUHP 2023) introduces a restorative justice orientation and recognises “tindakan” (treatment measures) as an alternative to punishment for offenders with mental disorders. However, Indonesia does not yet have a dedicated Mental Health Act governing forensic psychiatric diversion, nor specialised mental health courts, correctional psychiatric institutions, or embedded prison mental health teams. In this context, the development of a structured, tiered screening and risk stratification instrument is essential to operationalise the new legal framework, prevent harm, guide referral decisions, and support policy implementation amid substantial infrastructural limitations (11–14).

Therefore, there is an urgent need to develop a comprehensive mental disorder screening tool tailored to prisoners, designed for tiered use by correctional officers with varying educational levels. Such an instrument should be valid, reliable, and capable of detecting a broad spectrum of serious mental disorders while supporting decisions regarding placement, treatment, and rehabilitation, ultimately reducing both inmate risks and correctional officer burnout.

## Context for this study

Currently in Indonesia, *Direktorat Jenderal Pemasyarakatan* (or the Directorate General of Corrections), an implementing unit under the Ministry of Law and Human Rights responsible for formulating and executing correctional policies nationwide, is currently employs three mental health screening instruments across all correctional facilities. These instruments are as follows:

- Inmate Placement Screening Instrument (IPSI/*Instrumen Skrining Penempatan Narapidana*, ISPN): administered at the initial stage of incarceration to assess risks related to security, safety, institutional stability, and public safety (which may be influenced by underlying mental health conditions), thereby guiding inmate placement decisions.
- Inmate Rehabilitation Assessment System (IRAS/*Sistem Penilaian Pembinaan Narapidana*, SPPN): a periodic mental health screening tool designed to detect depression, anxiety disorders, psychosomatic conditions, malingering, suicide risk, and personality issues throughout incarceration.
- Indonesian Recidivism Risk Tool (IRR/*Risiko Residivisme Indonesia*, RRI): conducted prior to release to evaluate the risk of recidivism before inmates return to the community.

Among these, the IRAS/SPPN is the only instrument routinely used to evaluate inmates’ mental health and determine appropriate follow-up actions for those with psychiatric disorders. The instrument relies on correctional officers, particularly custodial supervisors, to evaluate inmate behavior. These officers serve not only as monitors but also as key assessors who, through observation, interviews, and document reviews, provide comprehensive evaluations of inmates across domains such as personality development, independence, attitudes, and mental health. The system enables structured and systematic monitoring, supported by CCTV, direct observation, and interviews during rehabilitation activities, thereby facilitating assessments that inform policy decisions. Beyond its evaluative role, the SPPN also functions as a behavioral control mechanism, as objective assessments may influence sanctions such as delaying parole or downgrading rehabilitation status, thus contributing to discipline and order within correctional facilities and encouraging greater inmate engagement in rehabilitation programs.

However, qualitative studies have identified several challenges in its implementation. Key obstacles include: a shortage of skilled professionals trained in the use of SPPN, such as psychologists and psychiatrists, leading to suboptimal assessment processes. Interviews with informants revealed that only a limited number of officers had received specialized training, resulting in incomplete or inconsistent behavioral observations. Additionally, low staff motivation and heavy workloads further undermine the effectiveness of rehabilitation efforts. Daily behavioral observations are also considered less reliable due to unclear assessment indicators, which often compromise objectivity. To optimize its function, the SPPN requires clearer standardized criteria and more comprehensive training to ensure accurate, objective, and meaningful evaluations that can support inmate rehabilitation.

**Figure 1:**
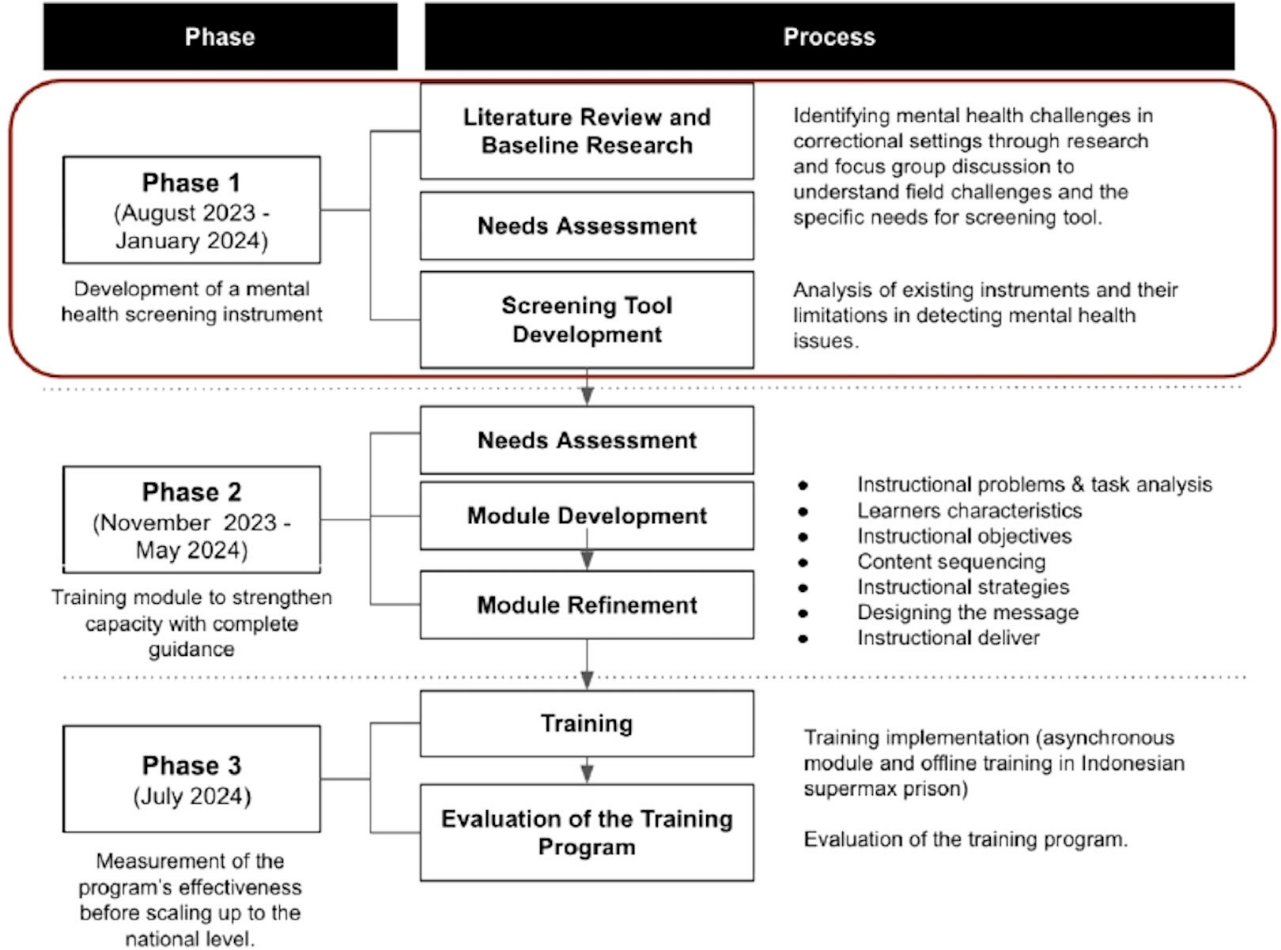
Phases of research project for the development of a screening tool for mental disorders among prisoners (this article reports only Phase 1).

The research team decided to seek funding for a project designed to develop screening tool(s) that are more appropriate, based upon the modification of the SPPN. This project has been designed to be completed in three phases (**Figure 1**). The current study was conducted from August 2023 to January 2024 as the first phase in a multiphasic research on screening for mental disorders in prisoners. **Figure 1** outlines all phases of the study; however, the present paper focuses exclusively on Phase 1. Findings from phase two and three will be reported in separate publications.

## Methods

### Development of instrument

This study represents Phase 1 of a multiphase mixed-methods instrument development and content validation project aimed at developing a structured mental health screening and risk stratification instrument for correctional settings in Indonesia. The methodological framework consisted of sequential stages including: (1) literature review to identify relevant psychiatric domains and existing correctional screening tools, (2) needs assessment survey among correctional and mental health stakeholders to identify operational gaps and contextual needs, (3) initial item generation and modification based on DSM-5 criteria and existing validated instruments, (4) Delphi-based expert consensus for content validation and refinement, and (5) development of risk stratification and management flowcharts for implementation in resource-limited prison settings.

*Literature review*. The first step of the tool development was to conduct a comprehensive literature review to determine: [1] mental disorders commonly found in prisoners, [2] commonly used screening instruments from different countries, and [3] best practices to be adapted and modified into the SPPN instrument currently used in Indonesia. PubMed and Google Scholar were searched using combinations of the following keywords: *mental disorder, screening, inmate, and instrument.*

*Needs assessment survey.* A survey was conducted to explore the current practices, challenges, and recommendations for improving the existing *Sistem Penilaian Pembinaan Narapidana* (SPPN) instrument, which currently serves as the standard for mental health screening among inmates in Indonesia. The survey aimed to capture the perspectives of professionals directly involved in correctional mental health management and rehabilitation, ensuring that the development of the new screening tool would address field-level needs and operational feasibility. A total of 39 respondents participated, representing a multidisciplinary group of practitioners and academics from four national hospital forensic psychiatry units, nine members from the Center for Detention Studies, eighteen representatives from the Directorate General of Corrections, one representative from the Indonesian Polytechnic of Corrections and Community Protection, six experienced probation officers, and one officer from the Human Resources Development Agency for Law and Human Rights. Participants were recruited using the snowball sampling technique to ensure broad professional coverage.

The survey questionnaire included open- and close-ended questions covering three main domains: (i) Current practices in conducting mental health screening and monitoring using the SPPN, (ii) challenges and limitations encountered during the implementation of SPPN (e.g., unclear indicators, lack of training, or workload constraints), and (iii) recommendations for improvement, including domains to be added, methods of assessment preferred, and suggestions for user-friendly design, risk stratification, and follow-up procedures. Prior to data collection, all participants were informed about the objectives and procedures of the study, and written informed consent was obtained from each respondent. Responses to open-ended questions were analyzed using thematic content analysis. Two investigators independently reviewed and coded the responses to identify recurring themes related to implementation challenges, operational needs, and recommendations for tool development. Differences in coding were resolved through discussion and consensus meetings within the research team. The themes presented in **Table 2** were derived inductively from patterns repeatedly identified across respondents’ narratives and recommendations.

### Expert feedback

Findings from the literature review and needs assessment survey were integrated to generate a preliminary list of screening tool items, which were subsequently distributed to a panel of experts using the Delphi technique. The inclusion criteria required respondents to: (i) have direct and diverse roles ranging from execution-level staff to policy decision-makers, thereby reflecting various viewpoints related to mental health issues in correctional settings; (ii) be willing to fully participate in the Delphi study; and (iii) possess a minimum of three years of experience in their respective work fields. The Delphi process consisted of three iterative rounds aimed at achieving consensus regarding the relevance, clarity, and operational feasibility of the proposed domains and items. In each round, experts received the updated list of items and rated each item on a 4-point Likert scale, ranging from 1 (very irrelevant) to 4 (very relevant). Items were accepted if at least 70% of experts rated them as 3 or 4, and if the median score reached ≥3.25. The 70% consensus threshold was predetermined based on commonly accepted standards in Delphi-based content validation studies. Experts were also invited to provide feedback on wording and to suggest additions or deletions of items. After each round, one investigator compiled scores and feedback, anonymized expert identifiers, and forwarded the dataset to the principal investigator for analysis. Revisions were made accordingly, including modification, merging, addition, or removal of items based on expert recommendations, and the updated list was redistributed for subsequent rounds. All invited experts participated throughout the Delphi process, resulting in no attrition across rounds. This iterative process continued until consensus was achieved and all items were accepted by the expert panel. The characteristics of the expert panel presented in **Table 1**.

**Table 1:** Characteristics of expert panel involved in item assessment.

No	Gender	Role	Institution
1	Female	Forensic Psychiatrist, Researcher and Scholar	National Referral Hospital
2	Male	Forensic Psychiatrist and Researcher	National Referral Hospital
3	Male	Deputy Director	Center for Detention Studies
4	Male	Lecturer of Correctional Science Study Program	Indonesian Polytechnic of Corrections and Community Protection
5	Male	Senior Policy Analyst	Directorate General of Corrections
6	Female	Junior Policy Analyst	Directorate General of Corrections
7	Male	Probation Officer	Directorate General of Corrections

## Results

### Construction and modification of initial items of the instrument

From the literature review, previous instruments such as Jail Screening Assessment Tool (JSAT), Generalized Anxiety Disorder-7 (GAD-7), SCL-90 Somatization Subscale, Modified Overt Aggression Scale (MOAS), Young Mania Rating Scale (YMRS), Positive and Negative Syndrome Scale (PANSS), Medication Adherence Rating Scale (MARS), were deemed to be appropriate for the aims of this study and served as theoretical foundation to modify or add items to the SPPN (2, 15–20). The SPPN only screens for depression, suicide risk, anxiety disorder, psychosomatic symptoms, and malingering. The tool does not assess more severe mental illnesses such as psychotic and manic symptoms, which may pose significant risks to both inmates and correctional staff. Thus, several items were adopted from the Positive and Negative Syndrome Scale (PANSS) and the Medication Adherence Rating Scale (MARS) and integrated into the current instrument. In addition, the history of psychiatric medication use was considered an important variable and therefore included in the instrument. A history of psychiatric medication use was included because it provides immediate clues about pre-existing mental health conditions and the likelihood of symptom relapse or instability during incarceration, also helps clinicians identify discrepancies between reported symptoms and past treatment, which is useful in assessing risk and detecting possible malingering.

The selection of domains was guided by three primary considerations: (1) prevalence and clinical urgency of psychiatric symptoms commonly encountered in correctional settings, (2) feasibility of identification by non-specialist correctional officers, and (3) potential immediate implications for institutional safety and emergency referral. Domains such as psychosis, suicide risk, aggression, and bipolar symptoms were prioritized due to their association with acute behavioral disturbances, self-harm, violence, and urgent psychiatric intervention needs.Several additional prison-relevant syndromes, including substance intoxication or withdrawal, trauma-related symptoms, cognitive impairment, delirium, and personality pathology, were considered during the conceptual phase but were not incorporated into the current instrument because of concerns regarding diagnostic complexity, overlap with medical assessment, limited feasibility for non-specialist screening, and the need to maintain operational simplicity. These domains remain important considerations for future expansion and validation phases.

Furthermore, our needs assessment survey proposed several key themes and identified needs related to the development of the new screening tool (**Table 2**). The result offered insights into the limitations of the current screening tools, including symptoms of some mental disorders that were not covered by the original SPPN and difficulties correctional and guardian officers face when using complex tools. The experts mentioned the need to clarify the assessment method for each item in the instrument, such as determining whether an item should be evaluated solely through interviews with inmates, through direct observation by officers, or by combining both approaches. They also emphasized the importance of establishing clearer classifications for certain items according to specific risk stratification levels, as well as developing a detailed emergency flowchart for managing suicide and aggression risks. To address these recommendations, the current instrument was refined by adopting the framework of the mhGAP Intervention Guide (mhGAP-IG), developed by the World Health Organization (WHO) and published in its second edition in 2016 (21). The mhGAP-IG provides practical, evidence-based guidance for identifying and managing common mental health conditions through simplified clinical assessments supported by follow-up algorithms. Building upon this framework, the instrument was enhanced to include structured risk stratification and an algorithmic approach to assessment and management, thus enabling correctional officers to develop clearer and more effective intervention plans. The initial findings led to an original list of 24 items for mental health screening among inmates.

**Table 2:** Key themes and needs identified from needs assessment survey.

Theme	Current challenge	Need identified for new tool
Continuity of assessment	The SPPN is applied at different stages without structured follow-up, and mental health domains are not consistently monitored.	A tool that enables continuous tracking of mental health symptoms across all stages, from intake to reintegration.
Staff capacity	Correctional and custodial officers lack specialized training and often do not know how to respond to inmate agitation or distress. Facilities and infrastructure are also limited.	Clear guidelines, simple language, and observable indicators that can be applied by staff with diverse educational and professional backgrounds.
Integration with existing systems	Data from current tools are not interconnected, resulting in incomplete reintegration information.	A tool that complements or modifies existing instruments (SPPN) and generates data that support reintegration planning.
Malingering and belief differentiation	Officers have difficulty distinguishing between malingering or true medical disorders.	Items that flag cases needing further assessment by trained professionals, with clear referral pathways.
Gender and case-specific risks	Risk factors and psychosocial needs vary widely (e.g., male vs. female inmates, high- vs. low-risk cases).	Flexibility to stratify risk levels and tailor interventions, including protocols for managing emergencies when inmates show potential violence toward self or others.

### Delphi method

Following the first Delphi round, 26 out of 30 items were approved by the expert panel, with median scores of 4 on a 4-point relevance scale (1 = very irrelevant to 4 = very relevant), reminding the reader of the scoring range. Items indicative of possible malingering of mental disorders were excluded from the instrument, as their evaluation necessitates a more comprehensive clinical assessment by qualified medical professionals. Additionally, several items within the depression, suicide, and aggression domains were collapsed due to redundancy identified by the expert panel. The panel further recommended the inclusion of new domains, namely bipolar, psychotic, and medication adherences. Items related to medication adherence were incorporated to ensure the continuity of essential treatments that support prisoners’ well-being within the correctional environment. Moreover, two items in the depression domain, namely the *feelings of guilt or worthlessness* and *weight change* were added in accordance with the diagnostic criteria for major depressive disorder outlined in the DSM-5. An additional item concerning *assaults and/or fights with officers or other inmates* was included in the aggression domain.

These revisions resulted in a list of 27 items. In Round 2, all 27 revised items, including the newly introduced domains of bipolar disorder, psychotic disorder, medication adherence, risk stratification, and the emergency management flowchart, were distributed to the expert panel. All 27 items achieved consensus with a median score of 4, and no further revisions were requested. A third round was subsequently conducted to allow experts to evaluate the complete, integrated instrument as a unified whole, as this was the first opportunity for the panel to assess all domains simultaneously. In the third round, all 27 items were endorsed without further modification (**Table 3**). The Delphi process was therefore concluded, as the final list met the predefined criteria.

**Table 3:** Overview of changes in diagnostic domains, item structure, and the addition of risk stratification and the assessment and management flowchart .

No. of initial item	Theme of initial item	Median			Feedback from panel of experts	Revision to the item	Theme of final item	No. of final item
		Round 1	Round 2	Round 3				
8	Depression				There were previously four items in the risk of suicide domain, but one item was removed (i.e., banging the head against a hard object), which represented nonsuicidal self-harm. The items were also rearranged in the domain of suicide risk based on their severity: (i) suicide attempt, (ii) self-harm, and (iii) suicidal ideation.	Refers to JSAT to stratify depression risk and initial management		7
D1	Low motivation	4	4	4	Modified after the first round to group symptoms included as low motivation	Rephrased and integrated several items regarding to DSM V criteria which is markedly diminished interest or pleasure in all, or almost all, activities most of the day	Low motivation	D2
D2	Sleep disturbance	4	4	4	Modified after the first round to include insomnia and hypersomnia	Refers to DSM V major depressive episode criteria which is Insomnia or hypersomnia nearly every day.	Sleep disturbance	D5
D3	Poor self care	4	–	–	Merge after the first round as symptoms Included as low motivation (D2)	–	–	–
D4	Appetite disturbance	4	4	4	Modified after the first round to include decrease or increase in appetite	Refers to DSM V major depressive episode criteria which is decrease or increase in appetite nearly every day	Appetite disturbance	D4
D5	Persistent low mood	4	4	4	Merge after the first round to group symptoms included as low mood	Refers to DSM V major depressive episode criteria which is depressed mood most of the day by either subjective report or observed by others	Persistent low mood and frequent tearfulness	D1
D6	Frequent tearfulness	4	–	–	Merge after the first round to group symptoms included as low mood (D1)	–	–	–
D7	Psychomotor retardation	4	4	4	Modified after the first round due to lack of clear criteria for symptoms included as psychomotor retardation	Refers to DSM V major depressive episode criteria which is Psychomotor agitation or retardation nearly every day	Psychomotor retardation	D6
D8	Social withdrawal	4	–	–	Merge after the first round as symptoms Included as low motivation (D2)	–	–	–
–	–	–	4	4	Added by the experts after first round regarding the importance to evaluate feelings of worthlessness or excessive or inappropriate guilt	Refers to DSM V major depressive episode criteria which is Feelings of worthlessness or excessive or inappropriate guilt nearly everyday	Feeling of guilt or worthlessness	D3
–	–	–	4	4	Added by the experts after first round, as indicator of depression, since significant weight change or appetite disturbance as the criteria of depression	Refers to DSM V major depressive episode criteria which is significant weight loss when not dieting or weight gain more than 5% of body weight in a month	Weight change	D7
4	Suicide					Refers to mhGAP Intervention Guide to stratify risk of suicide and initial management		3
B1	Self harm	4	4	4	Modified after the first round to specify behavior included as self harm	Further elaboration on description of self harm behaviour	Self harm	B2
B2	Banging head	4	–	–	Merge after the first round as self harm behaviour	–	–	–
B3	Suicide attempt	4	4	4	Modified after the first round to specify behaviour included as suicide attempt	Further elaboration on description of suicide attempt behaviour	Suicide attempt	B1
B4	Suicide ideation	4	4	4	Modified after the first round to elaborate how to explore suicide ideation	Further elaboration on how to explore suicide ideation using see, say, and signpost	Suicide ideation	B2
3	Anxiety				Two items in the domain of anxiety disorder were changed from repetitive behavior and afraid of being placed in a cell alone to excessive anxiety and worry, and feeling restless and keyed up on edge. These changes were made as repetitive behavior does not specifically represent anxiety disorder symptoms and overlaps with other diagnostic domains such as psychotic disorder and developmental disorder.	Refers to GAD-7 to stratify anxiety risk and initial management	Anxiety	3
C1	Repetitive behaviours	4	4	4	Modified after the first round due to the need to replace overlap with other psychiatric disorders (e.g., psychotic, developmental) with a clearer behavioral description: always appears tense and has difficulty relaxing.	Refers to DSM V general anxiety disorder criteria which is restlessness or feeling keyed up or on edge.	Feeling restless and keyed up on edge	C2
C2	Having trouble concentrating	4	4	4	Modified after the first round to group symptoms included as difficulty concentrating	Refers to DSM V general anxiety disorder criteria which is difficulty concentrating	Difficulty concentrating	C3
C3	Afraid of being placed in a cell alone	4	4	4	Modified after the first round as it was considered to focus more on phobic-type anxiety, rather than a general feature.	Refers to DSM V general anxiety disorder criteria which is Excessive anxiety and worry about a number of events or activities	Prone to excessive fear, worry, and/or anxiety	C1
1	Psychosomatic				The item criteria were further elaborated to specify situations in which psychosomatic symptoms may arise. These situations include: (i) specific activities (such as reading, exercise, etc.), (ii) participation in correctional activities, and (iii) encounters with correctional officers or certain inmates.	Refers to SCL-90 Somatization Subscale (Symptom Checklist-90) to stratify psychosomatic risk and initial management		1
P1	Experiencing somatic symptoms when exposed to stressful situations	4	4	4	Modified after the first round to provide further elaboration on symptoms and guidance for observing inmates.	Further elaboration of an item criteria of situation which increase physical symptoms	Psychosomatic	S1
7	Aggression				Initially, this domain included seven items, but four items were removed as they were deemed irrelevant to assessing aggression. Additionally, three items were collapsed into two: (iv) banging head against the wall and (v) speaking loudly, shouting, and displaying anger in the cell. To better assess potential risks, three new items were added: (i) assault or physical altercations with officers or inmates, (ii) threats of violence towards officers or inmates, and (iii) the use of offensive language or cursing directed at officers or inmates.	Refers to Modified Overt Aggression Scale (MOAS) to stratify aggression risk and initial management	Aggression	5
A1	Hitting the wall inside the room	4	4	4	Modified after the first round to further describe aggressive behavior involving the head, such as banging the head against the wall.		Aggressive behavior to self	A4
A2	Throwing objects inside the room	4	–	–	Merged after the first round into ‘Shouting or showing anger in cell ‘as both describe manifestations of outward-directed anger or agitation, thus combined into one broader behavioral item.	Further elaboration on description of aggression.	Shouting or showing anger in cell	A5
A3	Displaying anger in the room	4	–	–	Merge after the first round to avoid redundancy and to capture a broader range of aggressive or irritable behaviors	Further elaboration on description of aggression.	Shouting or showing anger in cell	A5
A4	Shouting inside the room	4	4	4	Modified after the first round to ‘shouting or showing anger in cell,’ as aggression is not always manifested through shouting alone.	Further elaboration on description of aggression.	Shouting or showing anger in cell	A5
A5	Damaging CCTV/other inventory in the room	4	–	–	Merge after the first round as threatens violence to officers or inmates	Further elaboration on description of aggression.	Threatens violence to officers or inmates	A2
A6	Shaking or kicking the room bars	4	–	–	Merge after the first round as threatens violence to officers or inmates	Further elaboration on description of aggression.	Threatens violence to officers or inmates	A2
A7	Climbing the room bars	4	–	–	Merge after the first round as threatens violence to officers or inmates	Further elaboration on description of aggression.	Threatens violence to officers or inmates	A2
–	–	–	4		Added by the experts after the first round regarding the importance of evaluating assaults and/or fights with officers or other inmates.	Further elaboration on description of violence to officers or inmates.	Assaults and/or fights with officers or other inmates	A1
0	Bipolar				Bipolar disorder was added in the diagnostic domain, which consisted of three items representing mania: (i) engaging in more activities than usual, (i) moving and/or talking more than usual, and the flight of ideas represented by (iii) having many ideas to talk about and/or quickly switching topics, making it hard for others to interrupt.	Refers toYoung Mania Rating Scale (YMRS) to stratify bipolar risk and initial management	Bipolar	3
–	–	4	4	4	Added at the first round to evaluate elevated goal-directed activity or psychomotor agitation as features of mania/hypomania	Refers to DSM V bipolar criteria which is increase in goal-directed activity	Increased goal-directed activity	I1
–	–	4	4	4	Added at the first round to evaluate excessive talking or difficulty being interrupted as features of mania/hypomania	Refers to DSM V bipolar criteria which is more talkative than usual or pressure to keep talking	Pressured speech	I2
–	–	4	4	4	Added at the first round to evaluate thought acceleration as features of mania/hypomania	Refers to DSM V bipolar criteria which is flight of ideas	Flight of ideas	I3
–	Psychotic				The psychotic domain was included with following items—(i) incoherent speech, (ii) aimless behavior, (iii) expression of irrational ideas, and (iv) presence of hallucinations was made to better capture the core symptoms of psychotic disorders.	Refers to Positive and Negative Syndrome Scale (PANSS) to stratify psychotic risk and initial management	Psychotic	4
–	–	4	4	4	Added at the first round to evaluate disorganized speech as feature of psychotic	Refers to DSM V psychotic disorders criteria which is disorganized speech	disorganized speech	P1
–	–	4	4	4	Added at the first round to evaluate disorganized behavior as feature of psychotic	Refers to DSM V psychotic disorders criteria which is disorganized behaviour	disorganized behavior	P2
–	–	4	4	4	Added at the first round to evaluate irrational ideas as feature of psychotic	Refers to DSM V psychotic disorders criteria which is irrational idea	irrational ideas	P3
–	–	4	4	4	Added at the first round to evaluate hallucination as feature of psychotic	Refers to DSM V psychotic disorders criteria which is hallucination	Hallucination	P4
	Medication adherence				This addition emphasizes the importance of psychiatric medication use that inmates may require, ensuring that necessary treatments are continued to maintain their mental well-being within the prison environment.	Refers to Medication Adherence Rating Scale (MARS) to stratify medication adherence and initial management		
–	–	4	4	4	Added at the first round to address the evaluation of medication non-adherence.	Addition of item medication non-adherence	Psychiatric and medical drug consumption history	O1
1	Malingering	4	–	–	The assessment of malingering requires a more elaborate and comprehensive clinical evaluation that goes beyond the capacity of a screening tool. Excluded after the first round as it is difficult for lay raters to evaluate reliably. Therefore, in cases where officers suspect malingering of mental disorder, they can immediately refer to medical professionals.	Referred to medical professionals	–	–
–	–	4	4	4	Added at the first round to stratify symptoms into mild, moderate, and severe categories, and incorporated corresponding management guidelines for each items	Refers to DSM V criteria and psychiatry emergency condition	Mild, moderate, and severe in each group	3
–	Assessment and Management Flowchat	–	4	4	Added at the first round to perform risk stratification and determine the appropriate management based on the level of emergency.	Refers to mhGAP Intervention Guide and Mental Health Service Guidelines for Prisoners and Detainees from the Ministry of Law and Human Rights and Directorate General of Corrections	Assessment and Management Flowchat	

To facilitate effective assessment, the tool integrates both observation and interview methods providing practical guidelines to correctional officers. During observation, officers evaluate inmate behavior as well as their responses to specific questions. Sample interview prompts are included to assist officers in collecting relevant information. The new instrument also specifies designated timeframes for both observations and interviews, aligned with relevant diagnostic criteria. Most assessments are conducted daily, with the exception of weight loss monitoring, which is performed monthly (21). The revisions in this instrument, which include changes to the diagnostic domains and item structure as well as the addition of risk stratification and the assessment and management flowchart, are presented in **Table 3**.

Given the limited availability of mental health professionals in correctional settings, correctional officers require not only symptom identification tools but also clear guidance on prioritizing cases and determining appropriate follow-up actions. Accordingly, revisions were made to the diagnostic domains and item structure, and a risk stratification system and an assessment and management flowchart were incorporated.

Risks are classified into three levels: mild (green color), moderate (yellow), and severe (red) (**Table 4**). Management strategies are tailored to the level of risk, with mild cases typically managed through counseling by trained correctional officers, whereas moderate and severe cases require referral to a trained professional, such as a general practitioner or psychiatrist. Once the inmate has been stratified by risk level, correctional staff continue daily evaluations to monitor for potential changes in classification (22).

**Table 4:** Final domains, items, methods of screening, and risk stratification of the screening tool.

Domain	No.	Item	Methods		Frequency
			Observation	Interview	
Depression	D1	Depressed mood	-✓		1x/day
	D2	Markedly diminished interest in activities	✓	✓	1x/day
	D3	Excessive feelings of worthlessness/guilt	–	-✓	1x/day
	D4	Dietary changes	-✓	✓	1x/day
	D5	Sleep changes	✓	-✓	1x/day
	D6	Slowed speech	-✓	✓	1x/day
	D7	Weight loss		-✓	1x/month
Risk of suicide	B1	Suicide attempt	✓		1x/day
	B2	Self-harm	✓		1x/day
	B3	Suicidal ideation		✓	1x/day
Anxiety	C1	Excessive fear and/or worry and/or anxious	✓	–	1x/day
	C2	Feeling restless	-✓		1x/day
	C3	Inability to maintain focus	✓		1x/day
Psychosomatic disorder	S1	Physical symptoms that appear on certain situations	-✓	✓	1x/day
Aggression	A1	Assaults and/or fights with officers or other inmates	✓		1x/day
	A2	Threatens violence towards officers or other inmates	-✓		1x/day
	A3	Curses or insults officers or other inmates	✓	–	1x/day
	A4	Banging head to the wall	-✓		1x/day
	A5	Shouting or showing anger in cell	✓	–	1x/day
Bipolar disorder	I1	Do more activities than usual	✓	✓	1x/day
	I2	Move and/or talk more than usual	-✓		1x/day
	I3	Flight of ideas	✓		1x/day
Psychotic disorder	P1	Incoherent speech	-✓		1x/day
	P2	Purposeless behavior	✓		1x/day
	P3	Distorted cognitive pattern	-✓		1x/day
	P4	Hallucination	✓		1x/day
Psychiatric and medical drug consumption history	O1	Medication non-adherence	-✓		1x/day

### Screening tool development

The previously available observer-rated screening tool, SPPN, resulted in categorizations into three main classifications: (i) assessment of counselling and personality, (ii) behavior assessment, and (iii) mental health assessment. There were six domains in the observer-rated SPPN including: (i) depression; (ii) suicide risk; (iii) anxiety; (iv) psychosomatic disorder; and (v) malingering. Based on the DSM-5 criteria, we classified aggression into mental health assessment as it is domain (15). After review and analysis, there were gaps in the domains previously used. Several changes were made and three domains were added to the newly developed screening tool: psychotic disorder, bipolar disorder and history of psychiatric medication use. All items within the domains were rephrased and underwent risk stratification, with some items removed because they did not adequately represent the diagnostic domain (**Table 3**).

To facilitate effective assessment, the tool incorporates both observation and interview methods as guidelines for correctional officers. During the observation process, officers assess both the inmates’ behavior and their responses to specific questions. Sample interview questions are provided to guide officers in gathering relevant information. The new instrument also includes designated timeframes for observations and interviews, aligned with relevant diagnostic criteria. Most observations and interviews are conducted on a daily basis, with the exception of weight loss monitoring, which is conducted monthly (23).

Risk stratification and initial management based on the risks were also added. The risks were classified into mild, moderate, and severe (**Table 4**). The management is based on the stratified risks with each category involving different referrals. The mild severity risk does not necessitate structured risk management because their symptoms do not pose immediate safety concerns or functional impairment, while moderate and severe risks involve a trained professional, such as a general practitioner or psychiatrist. Once the inmate is stratified based on their risk, the correctional guardian will further evaluate daily for a possibility of change in the risk stratification (22).

Risk stratification for the domains mentioned above is divided into mild (green), moderate (yellow), and severe (red), with tailored interventions corresponding to each category and domain. The current stratification thresholds were developed based on expert consensus and adaptation from existing psychiatric emergency and correctional mental health frameworks. Therefore, these thresholds should be considered preliminary and will require further empirical validation in subsequent phases of the study, particularly for suicide and aggression-related pathways. For psychotic and psychiatric or medical drug consumption history domains, the presence of any symptoms for any duration will be classified as severe, which requires immediate further assessment by general practitioners for assessment and management for possibility of referral to psychiatrist. The presence of any symptoms in the risk of suicide domain will be classified as moderate or severe and requires further assessment by trained professional (psychologist or general practitioner) to investigate and manage the condition. As for the remaining domains, which are depression, anxiety, psychosomatic, aggression, and bipolar disorder, stratification is based on the number of identified symptoms which must be observed for a specified number of days vary according to the symptoms.

### Initial management flowchart of psychiatric emergency

**Figure 2:**
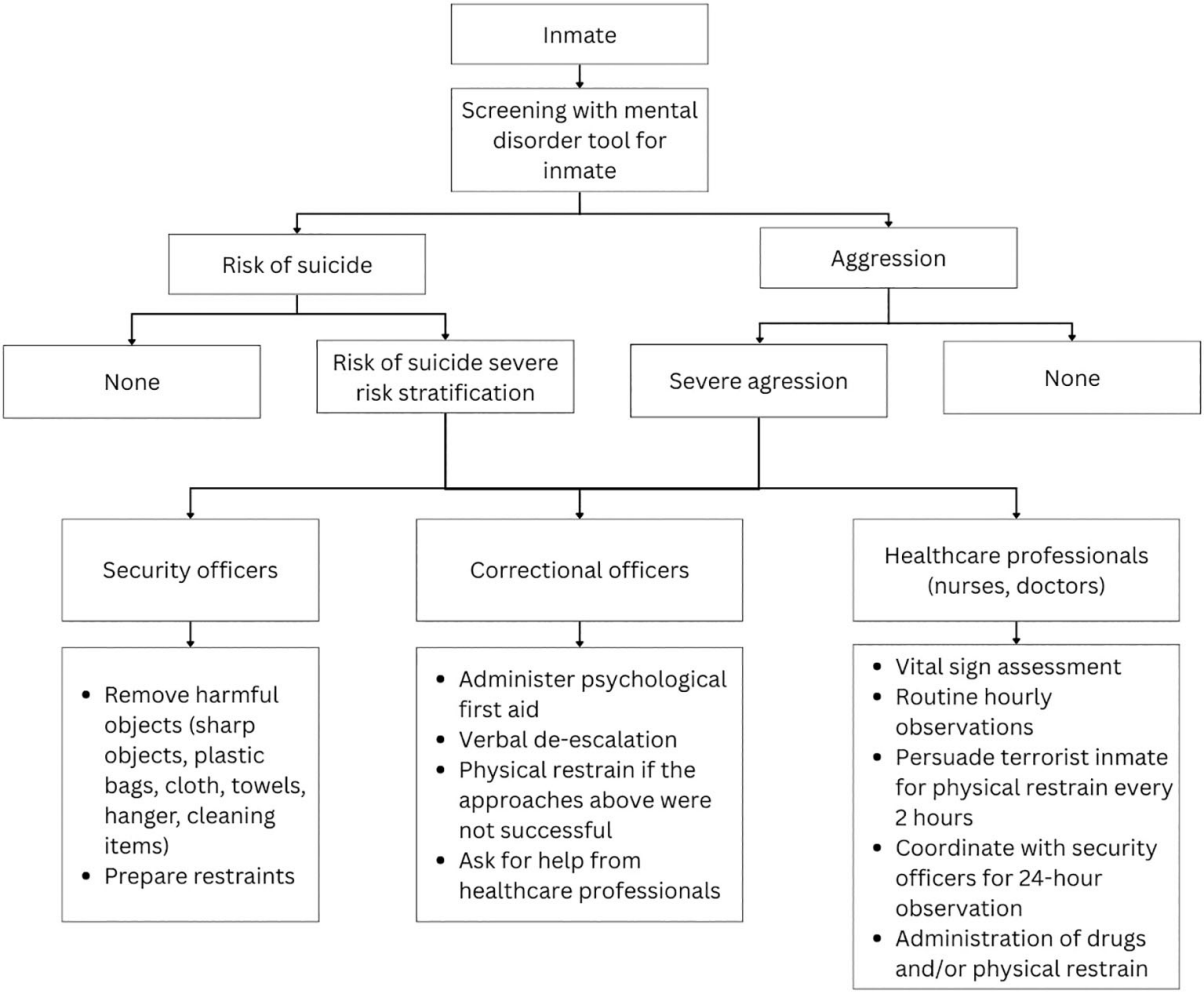
Flowchart of emergency for risk of suicide and aggression.

In cases where screening of the prisoners revealed risk of suicide or aggression, immediate initial management is required. The personnel involved in the initial management are security officers to maintain security and safety; correctional officers as the frontline screen and manage the prisoner through psychological first aid, verbal de-escalation and physical restraint if needed; healthcare professionals such as nurses and doctors assess vital signs, administer medication, and are the next step of management. **Figure 2** shows the details of the flowchart for psychiatric emergencies in prisoners.

## Discussion

The present paper reports only the first phase of the larger multiphase instrument development project, focusing primarily on item generation and content validation aimed to develop a standardized screening tool for early detection of mental health problems among prisoners in Indonesia by modifying and expanding the existing *Sistem Penilaian Pembinaan Narapidana* (SPPN) instrument. The SPPN has been widely used in Indonesian correctional settings as part of the Inmate Rehabilitation Assessment System (IRAS) to monitor inmates’ progress and behavioral development. While the instrument provides a structured approach for correctional officers to evaluate inmates, previous studies and qualitative evaluations have indicated significant limitations in its sensitivity and coverage of psychiatric symptoms, particularly severe mental illnesses such as psychotic and bipolar disorders (24–28). Furthermore, its reliance on subjective observation and insufficiently trained raters has been associated with inconsistent scoring and under-detection of inmates requiring psychiatric referral (7).

The modification of the SPPN in this study followed international best practices in instrument development through adaptation of existing tools, rather than constructing a completely new one. This approach aligns with several previous studies that demonstrated the advantages of using validated instruments as the foundation for context-specific adaptations. In the current study, the development process integrated domains from six widely used screening tools, the Jail Screening Assessment Tool (JSAT), Generalized Anxiety Disorder-7 (GAD-7), Symptom Checklist-90 (SCL-90) Somatization Subscale, Modified Overt Aggression Scale (MOAS), Young Mania Rating Scale (YMRS), and Medication Adherence Rating Scale (MARS). The resulting eight-domain, 27-item screening tool retained the structure of the original SPPN but expanded its diagnostic scope and introduced risk stratification and flowchart-based management to facilitate decision-making by correctional officers. Comparable methodologies have been reported in previous research developing mental health screening tools through the modification of existing frameworks (17). A previous study on the development and validation of a comprehensive scale to measure various dimensions of customer aggression used a multi-phase methodological approach to ensure the scale’s validity and reliability (29).

The present findings underscore that instrument modification must prioritize usability for non-specialist staff who serve as frontline assessors in correctional settings. On the needs assessment survey, experts emphasized the need for clear behavioral anchors, simple operational language, and tiered management instructions that can be followed by officers with diverse educational backgrounds. The integration of both observation and interview-based assessments into the tool was therefore essential to ensure feasibility, as similar dual-modality approaches have been recommended in studies developing correctional mental health instruments (2, 23). The introduction of risk stratification levels (mild, moderate, severe) and the accompanying management flowchart was another innovative modification inspired by prior frameworks such as the WHO mhGAP Intervention Guide (2016) and the STAIR Model (Screening, Triage, Assessment, Intervention, and Reintegration) proposed by Simpson et al. (2013) (2). Both models advocate structured, tiered responses to mental health crises in low-resource correctional settings. By adopting these principles, the newly developed tool offers not only detection but also initial management guidance, addressing the actionability gap identified in previous evaluations of SPPN.

From a theoretical perspective, this study contributes to the growing evidence that adaptation and contextualization of existing instruments are essential in resource-limited or specialized populations. Although psychometric strength is essential, it must be balanced with practical utility. Instruments that are overly complex or require specialized interpretation often demonstrate limited effectiveness in real-world settings. For a measure to possess true clinical utility, it should not only demonstrate validity and reliability but also be simple, time-efficient, easily interpretable, and capable of providing meaningful information to support decision-making. Recent evidence further underscores the importance of evaluating an instrument’s usability to guide iterative refinement and enhance its applicability across diverse contexts and user populations. (22, 30)

The newly developed instrument was deliberately designed to maintain high content validity confirmed through expert consensus (Delphi technique) while remaining operationally simple for correctional officers to apply in daily practice. At this stage, the study establishes content validity through expert consensus using the Delphi technique, but additional psychometric evaluation remains necessary. Future phases will examine construct validity, criterion validity, inter-rater reliability, test-retest reliability, and implementation feasibility under real correctional conditions. In addition, the instrument has not yet undergone formal field validation in real prison environments; therefore, important practical considerations such as administration time, training requirements, usability in overcrowded facilities, consistency between users, and acceptability among correctional staff remain to be evaluated. Despite these limitations, the present study represents an important first step toward strengthening mental health screening within Indonesian correctional settings by providing a structured, context-specific instrument that expands the existing SPPN framework and supports early identification and management of priority mental health conditions among inmates.

Despite these limitations, this project represents an important step toward improving the mental health screening system within Indonesia’s correctional institutions. By modifying the existing SPPN framework, the new instrument leverages familiar operational structures, reduces training demands, and provides a standardized approach to detect and manage inmates at risk of mental disorders. Its development aligns with Indonesia’s national correctional health policy emphasizing early detection, interprofessional collaboration, and reintegration-oriented rehabilitation (31).

## Conclusion

A structured screening tool to guide correctional officers in identifying mental disorders among prisoners is essential for referral to mental health services within prison settings. We developed an eight-domain screening tool comprising 27 items accompanied by risk stratification, guidance on decision making and initial management, specifically designed to screen for selected priority mental health syndromes and psychiatric emergencies commonly encountered in prison settings. The tool is accompanied by guidelines for data collection, including observations and interview questions. As the first phase of a multistep research project, this tool aims to facilitate improved delivery of mental health services in Indonesian prison settings.

## Data Availability

The raw data supporting the conclusions of this article will be made available by the authors, without undue reservation.

## References

[1] Sarma KM Carthy SL Cox KM . Mental disorder, psychological problems and terrorist behaviour: a systematic review and meta‐analysis. Campbell Syst Rev. (2022) 18. doi: 10.1002/cl2.1268 36911352 PMC9186052

[2] Steadman HJ Osher FC Robbins PC Case B Samuels S . Prevalence of serious mental illness among jail inmates. Psychiatr Serv. (2009) 60:761–5. doi: 10.1176/ps.2009.60.6.761 19487344

[3] . Directorate General of Corrections, Ministry of Law and Human Rights (2023).

[4] Wirya A Permata A . Kematian Tahanan, Kegagalan Pemidanaan. Jakarta Selatan: Lembaga Bantuan Hukum Masyarakat (2017).

[5] Felson RB Silver E Remster B . Mental disorder and offending in prison. Crim Justice Behav. (2012) 39:125–43. doi: 10.1177/0093854811428565

[6] Gilling McIntosh L Rees C Kelly C Howitt S Thomson LDG . Understanding the mental health needs of Scotland’s prison population: a health needs assessment. Front Psychiatry. (2023) 14. doi: 10.3389/fpsyt.2023.1119228 37265556 PMC10229789

[7] Simpson AIF Gerritsen C Maheandiran M Adamo V Vogel T Fulham L . A systematic review of reviews of correctional mental health services using the STAIR framework. Front Psychiatry. (2022) 12. doi: 10.3389/fpsyt.2021.747202 35115956 PMC8806032

[8] Laporan Penelitian: Baseline Riset Program Penguatan Kapasitas Petugas Pemasyarakatan dalam Penanganan Warga Binaan dan Klien Teroris melalui Pendekatan Psikoedukasi. Jakarta: Ditjenpas (2023).

[9] Darani SA McMaster R Wolff E Bonato S Simpson A Glancy G . Addressing the mental health needs of inmates through education for correctional officers—a narrative review. J Continuing Educ Health Professions. (2023) 43:247–53. doi: 10.1097/ceh.0000000000000484 36988450 PMC10664778

[10] Waloeya SD Rahayu M . Implementasi kebijakan sistem penilaian pembinaan narapidana (SPPN) di lapas perempuan kelas IIA Tangerang. Jurnal Intelektualita: Keislaman Sosial Dan Sains. (2023) 12. doi: 10.19109/intelektualita.v12i002.19803

[11] Undang-undang republik Indonesia nomor 1 tahun 2023 tentang kitab undang-undang hukum pidana (KUHP). In: Lembaran Negara Republik Indonesia Tahun 2023 Nomor 1; Tambahan Lembaran Negara Republik Indonesia Nomor 6842, Jakarta.

[12] Rinaldi F . Implementation of restorative justice in criminal case settlement to overcome over capacity of correction institutions. Cessie: Jurnal Ilmiah Hukum. (2025) 4:1–10. doi: 10.55904/cessie.v4i2.1647

[13] Sihombing LA Nuraeni Y Komarudin K Karunia K . Restorative justice as a new breakthrough to reduce recidivism and promote reform in the criminal law system. Jurnal USM Law Rev. (2025) 8:441–53. doi: 10.26623/julr.v8i1.11807

[14] Fazacholil MG Saefudin Y . Adaptive criminal liability in national legal reform: accommodating mental disability in criminal law. Neoclassical Legal Review: J Law Contemp Issues. (2025) 4:52–9. doi: 10.32734/nlrjolci.v4i2.20658

[15] American Psychiatric Association . The Diagnostic and Statistical Manual of Mental Disorders, 5th Edition. 5th ed. American Psychiatric Publishing, Inc (2013).

[16] Wei Y McGrath PJ Hayden J Kutcher S . Measurement properties of tools measuring mental health knowledge: a systematic review. BMC Psychiatry. (2016) 16:297. doi: 10.1186/s12888-016-1012-5 27553955 PMC4995619

[17] McHugh ML . Interrater reliability: the kappa statistic. Biochem Med (Zagreb). (2012) 22:276–82. doi: 10.11613/bm.2012.031 PMC3900052 23092060

[18] Aji Dimas Pangestu M Muhammad Politeknik Ilmu Pemasyarakatan A . Analisis faktor the Brief Jail Mental Health Screen versi Bahasa Indonesia dengan metode confirmatory factor analysis (CFA). In: Jurnal Ilmu Sosial Dan Humaniora, vol. 5. DenpasarL Jayapangus Press (2022). Available online at: https://jayapanguspress.penerbit.org/index.php/ganaya424 (Accessed June, 2025).

[19] Evans C Brinded P Simpson AI Frampton C Mulder RT . Validation of brief screening tools for mental disorders among New Zealand prisoners. Psychiatr Serv. (2010) 61:923–8. doi: 10.1176/ps.2010.61.9.923 20810592

[20] Graf M Wermuth P Häfeli D Weisert A Reagu S Pflüger M . Prevalence of mental disorders among detained asylum seekers in deportation arrest in Switzerland and validation of the Brief Jail Mental Health Screen BJMHS. Int J Law Psychiatry. (2013) 36:201–6. doi: 10.1016/j.ijlp.2013.04.009 23642321

[21] Grisso T . Jail Screening Assessment Tool (JSAT): guidelines for mental health screening in jails. Psychiatr Serv. (2006) 57:1049–50. doi: 10.1176/appi.ps.57.7.1049-a 8723190

[22] Prasetio CE Triwahyuni A Prathama AG . Psychometric properties of Self-Report Questionnaire-20 (SRQ-20) Indonesian version. Jurnal Psikologi. (2022) 49:69. doi: 10.22146/jpsi.69782

[23] Budikayanti A Larasari A Malik K Syeban Z Indrawati LA Octaviana F . Screening of generalized anxiety disorder in patients with epilepsy: using a valid and reliable Indonesian version of Generalized Anxiety Disorder-7 (GAD-7). Neurol Res Int. (2019) 2019:1–10. doi: 10.1155/2019/5902610 31275648 PMC6582805

[24] Martin MS Colman I Simpson AI McKenzie K . Mental health screening tools in correctional institutions: a systematic review. BMC Psychiatry. (2013) 13:275. doi: 10.1186/1471-244x-13-275 24168162 PMC4231452

[25] Okamura M Okada T Okumura Y . Recidivism among prisoners with severe mental disorders. Heliyon. (2023) 9:e17007. doi: 10.1016/j.heliyon.2023.e17007 37484360 PMC10361118

[26] Gonzales L Kois LE Chen C López-Aybar L McCullough B McLaughlin KJ . Reliability of the term “serious mental illness”: a systematic review. Psychiatr Serv. (2022) 73:1255–62. doi: 10.1176/appi.ps.202100661 35895839

[27] Merola LM Vovak H . The challenges of terrorist and extremist prisoners. Crim Justice Policy Rev. (2013) 24:735–58. doi: 10.1177/0887403412463048

[28] Boberg M Jeppesen U Arnfred S Nordgaard J . Do we know the mind of others? Suspicion of Malingering in emergency psychiatry. Nord J Psychiatry. (2023) 77:234–9. doi: 10.1080/08039488.2022.2083676 35714972

[29] Dian CN Effendy E Amin MM . The validation of Indonesian version of Patient Health Questionnaire-9. Open Access Maced J Med Sci. (2022) 10:193–8. doi: 10.3889/oamjms.2022.9293

[30] Oktaviana M Wimbarti S . Validasi klinik strenghts and difficulties questionnaire (SDQ) sebagai instrumen skrining gangguan tingkah laku. 41:.

[31] Kubiak S Comartin EB Hanna J Swanson L . Identification, referral, and services for individuals with serious mental illness across multiple jails. J Correctional Health Care. (2020) 26:168–82. doi: 10.1177/1078345820920703 32390543

